# The measurement of KRAS G12 mutants using multiplexed selected reaction monitoring and ion mobility mass spectrometry

**DOI:** 10.1002/rcm.8657

**Published:** 2020-02-14

**Authors:** Rachel L Norman, Rajinder Singh, James I. Langridge, Leong L. Ng, Donald J.L. Jones

**Affiliations:** ^1^ Leicester Cancer Research Centre, Leicester Royal Infirmary University of Leicester Leicester LE1 5WW UK; ^2^ Waters Corporation, Wilmslow SK9 4AX UK; ^3^ Department of Cardiovascular Sciences University of Leicester and National Institute for Health Research Leicester Biomedical Research Centre Glenfield Hospital Leicester LE1 7RH UK

## Abstract

**Rationale:**

There is a considerable clinical demand to determine key mutations in genes involved with cancer which necessitates the deployment of highly specific and robust analytical methods. Multiplex liquid chromatography with selected reaction monitoring (LC/SRM) assays offer the ability to achieve quantitation down to levels expected to be present in clinical samples. Ion mobility mass spectrometry (IMS/MS) assays can provide increased peak capacity and hence separation in an extremely short time frame, and in addition provide physicochemical data regarding the collision cross‐section of an analyte which can be used in conjunction with the *m*/*z* value of an ion to increase detection specificity.

**Methods:**

For LC/SRM, unlabelled peptides and corresponding stable‐isotope‐labelled standards were spiked into digested human plasma and analysed using ultrahigh‐performance liquid chromatography (UHPLC) coupled to a triple quadrupole mass spectrometer to enable the generation of analyte‐specific calibration lines. Synthetic unlabelled peptides were infused into a Synapt G2 mass spectrometer for travelling wave ion mobility separation and ^TW^CCS_N2_ values were derived from comparison with previously generated ^TW^CCS_N2_ calibration values.

**Results:**

Linear calibration lines (0.125 to 25 fmol/μL) were established for each of the KRAS peptides. UHPLC separated the peptides and hence enabled them to be split into different retention time functions/windows. This separation enabled detection of three or four transitions for each light and heavy peptide with at least 10 points per peak for accurate quantitation. All six KRAS G12 peptides were separated using IMS/MS, enabling precise ^TW^CCS_N2_ values to be determined. Although some of the G12 peptides chromatographically co‐eluted, all the peptides were distinguished by *m*/*z*, retention time and/or drift time.

**Conclusions:**

This study advocates that LC/SRM and IMS/MS could both be used to identify single amino acid substitutions in KRAS as an alternative to commonly used methods such as circulating tumour DNA analysis.

## 1 INTRODUCTION

Targeted liquid chromatography with selected reaction monitoring (LC/SRM) assays have great potential for use in the clinic to detect protein biomarkers for the diagnosis and monitoring of a wide variety of diseases. Despite offering many advantages, significant challenges still exist and a very limited number of LC/SRM‐based assays have been adopted in the clinical setting to date.^1^ Implementing this technique in clinical laboratories necessitates the development of robust, quantitative assays for clinically relevant biomarkers for which reliable detection methods do not currently exist.^2^ In addition, if the assay is unable to provide a financial operation cost advantage, the information derived from the assay has to be of superior quality to that provided by other methods.

KRAS is a protein first identified in the Kirsten rat sarcoma virus^3^ and is involved in cell signalling which activates cell growth in response to growth factor binding. It contains an intrinsic GTPase which switches off the pathway; however, single amino acid substitutions can inactivate the GTPase. This leads to constitutive activation of KRAS and aberrant growth. KRAS is implicated in many cancers including colorectal cancer, pancreatic cancer and non‐small cell lung cancer.^4^, ^5^, ^6^ Amino acid substitutions are driver mutations, occurring early in the carcinogenesis process.^7^ Thus, methods that identify KRAS mutations could enable detection of early‐stage cancer and subsequently inform the clinician of preferential treatment regimens. KRAS mutations occur at positions 12, 13 and 61 of the KRAS gene with substitutions of the glycine at position 12 the most common.^8^, ^9^ KRAS has an extremely low copy number and has not previously been detected in human plasma. Consequently, determination of KRAS status is primarily undertaken using genetic testing.^10^ However, genetic testing relies on invasive, costly and time‐consuming (typically around 7 days) methodologies which rely on high‐fidelity amplification to achieve genetic determination.

Travelling wave ion mobility separation (TWIMS) enables gas‐phase electrophoretic separation by creating a dynamic pulse of alternating voltages that results in a “travelling wave” of ions within a dense gas‐filled chamber (in this case nitrogen).^11^ Ions are separated by their size, shape or charge state. Since mutated peptides differ by a single amino acid and some of them co‐elute using reversed‐phase LC/SRM, we also investigated the use of TWIMS to improve separation of peptides and determine the collision cross‐section (CCS) values, thus potentially allowing the use of this parameter to specifically identify the mutations in biological samples. Ion mobility separates ions in milliseconds, nesting between ultra high‐performance liquid chromatography (UHPLC) which separates in seconds and time of flightwhich separates on a timescale of microseconds, resulting in increased peak capacity and offering the ability to analyse complex samples in a very short amount of time which could be advantageous in the clinical setting. Peptides unique to each RAS protein were selected to distinguish between the RAS isoforms, NRAS, HRAS and KRAS, because the tryptic peptide containing the mutation is found in all isoforms. Stable‐isotope‐labelled (heavy) standards were incorporated into the LC/SRM assay to enable absolute quantification of the peptides using isotope dilution and multipoint calibration lines.

## 2 MATERIALS AND METHODS

### 2.1 Materials

All chemicals and reagents were purchased from Sigma Aldrich (Poole, UK) or Fisher Scientific (Loughborough, UK) unless otherwise stated. Stable‐isotope‐labelled (heavy) peptides were purchased from Pepscan (Lelystad, The Netherlands) and unlabelled (light) custom‐synthesised peptides were acquired from Genecust (Ellange, Luxembourg).

### 2.2 Liquid chromatography

For LC/SRM analysis, samples were injected onto a NanoAcquity UHPLC system with a 2G‐V/M Symmetry C18 trap column (180 μm × 20 mm, 5 μm) for desalting and chromatographic focusing before elution onto an Acquity HSS T3 analytical UHPLC column (75 μm × 250 mm, 1.8 μm) (all from Waters, Milford, MA, USA). The analytical column temperature was set at 40°C and the auto sampler temperature was maintained at 6°C. Trapping occurred for 3 min with 99.9% solvent A and 0.1% solvent B at a flow rate of 5 μL/min. A 60 min liquid chromatography gradient was initiated on elution from the trap column. The following gradient was used: 0 min, 3% B; 40 min, 50% B; 40.33 min, 85% B; 51.60 min, 85% B; and 52 min, 3% B. The flow rate was set at 0.3 μL/min. Solvent A was LC/MS‐grade water containing 0.1% formic acid. Solvent B was acetonitrile containing 0.1% formic acid.

### 2.3 Mass spectrometry

A Waters Xevo TQ mass spectrometer was operated in positive electrospray ionization mode. The capillary voltage was set at 2.40 kV and the cone voltage was set at 30 V. Argon was used as the collision gas and the collision energy was optimized for each peptide by repeated injections using different collision voltages. Initially the precursor ion for each peptide was determined by performing a mass spectrometry (MS) scan followed by a product ion scan to identify the most abundant product ions to select as the SRM transitions. Exact *m*/*z* values were extracted from the raw data using Skyline software (MacCoss Laboratory).^12^ Tryptically digested peptides at residues 89–97 are unique to each RAS protein and were used to distinguish between KRAS, HRAS and NRAS. The RAS‐specific peptide sequences were SFEDIHQYR, SFEDIHHYR and SFADINLYR for HRAS, KRAS and NRAS, respectively. The RAS G12 tryptically digested peptide spanned residues 6–16 (LVVVGAGGVGK). SRM transitions and optimized collision energies for each peptide are presented in Table 1. Human volunteer plasma (25 μL) was added to 275 μL of 50 mM ammonium bicarbonate, pH 7.8. Dithiothreitol was added to a final concentration of 15 mM and incubated for 30 min at 60°C. Iodoacetamide was added to a final concentration of 20 mM and incubated for 30 min in the dark at room temperature. Then, 8 μL of 5 μg/μL trypsin (dissolved in ammonium bicarbonate, pH 7.8) was added and the sample was left overnight at 37°C. On the next day, 3 μL of formic acid was added to stop trypsinolysis. The sample was freeze‐dried overnight, then reconstituted in 1 mL of 0.1% formic acid and acetonitrile (97:3), and split into 100 μL aliquots and stored at −80°C. Calibration lines were generated by spiking into 20 μL of the digested plasma the nine peptide standards ranging from 0.125 to 25 fmol/μL and 25 fmol/μL of the equivalent heavy‐labelled standard.

**Table 1:** Optimized SRM transitions for G12 and Ras‐specific peptides

Peptide	Protein	Precursor *m*/*z* (light)	Product *m*/*z* (light)	Precursor *m*/*z* (heavy)	Product *m*/*z* (heavy)	Collision energy (eV)
SFEDIHQYR	HRAS	398.8561	302.1535	402.1922	307.1577	15
SFEDIHQYR		398.8561	358.6956	402.1922	363.6997	15
SFEDIHQYR		398.8561	480.7303	402.1922	485.7345	15
SFEDIHHYR	KRAS	401.8562	363.1957	405.1923	368.1998	15
SFEDIHHYR		401.8562	475.2412	405.1923	485.2495	15
SFEDIHHYR		401.8562	558.7647	405.1923	563.7688	15
LVVVGAGGVGK	Ras WT	478.3004	372.2241	482.3075	376.2312	17
LVVVGAGGVGK		478.3004	545.3042	482.3075	553.3184	17
LVVVGAGGVGK		478.3004	644.3726	482.3075	652.3868	17
LVVVGAGGVGK		478.3004	743.441	482.3075	751.4552	17
LVVVGAAGVGK	Ras G12A	485.3082	502.2984	489.3153	510.3126	18
LVVVGAAGVGK		485.3082	559.3198	489.3153	567.334	18
LVVVGAAGVGK		485.3082	658.3882	489.3153	666.4024	18
LVVVGAAGVGK		485.3082	757.4567	489.3153	765.4709	18
LVVVGASGVGK	Ras G12S	493.3057	387.2294	497.3128	391.2365	17
LVVVGASGVGK		493.3057	575.3148	497.3128	583.3289	17
LVVVGASGVGK		493.3057	674.3832	497.3128	682.3974	17
LVVVGASGVGK		493.3057	773.4516	497.3128	781.4658	17
LVVVGAVGVGK	Ras G12V	499.3239	587.3511	503.331	595.3653	18
LVVVGAVGVGK		499.3239	686.4196	503.331	694.4338	18
LVVVGAVGVGK		499.3239	785.488	503.331	793.5022	18
LVVVGADGVGK	Ras G12D	507.3031	546.2882	511.3102	554.3024	18
LVVVGADGVGK		507.3031	603.3097	511.3102	611.3239	18
LVVVGADGVGK		507.3031	702.3781	511.3102	710.3923	18
LVVVGADGVGK		507.3031	801.4465	511.3102	809.4607	18
LVVVGAC[+57]GVGK	Ras G12C	529.805	591.2919	533.8121	599.3061	19
LVVVGAC[+57]GVGK		529.805	648.3134	533.8121	656.3276	19
LVVVGAC[+57]GVGK		529.805	747.3818	533.8121	755.396	19
LVVVGAC[+57]GVGK		529.805	846.4502	533.8121	854.4644	19
SFADINLYR	NRAS	549.7282	421.1718	554.7867	421.1718	19
SFADINLYR		549.7282	565.3093	554.7867	575.3175	19
SFADINLYR		549.7282	793.4203	554.7867	803.4285	19
SFADINLYR		549.7282	864.4574	554.7867	874.4657	19

A 500 fmol/μL mixture of wild‐type and five mutant KRAS 6–16 peptides was mixed in a ratio of 1:1 with the digested plasma solution described above and infused into a Waters Synapt G2 mass spectrometer at a flow rate of 1 μL/min. The instrument was operated in positive ion electrospray mode. The capillary voltage was set at 2.40 kV and the cone voltage to 35 V. The flow rate of the nitrogen in the ion mobility separation (IMS) cell was 111 mL/min and the flow rate of helium in the helium cell was 180 mL/min. Different wave velocities were tested from 500 to 1400 m/s at 100 m/s intervals to obtain the optimum orthogonal separation. A wave velocity of 1330 m/s was ultimately chosen for maximal separation. For each sample, data were acquired for 5 min to improve the signal‐to‐noise ratio.

### 2.4 CCS determination

A bovine serum albumin (BSA) digest standard (Waters Ltd, Elstree, UK) was analysed using a data‐independent acquisition method which incorporates TWIMS which is known as high‐definition MS^E^ (HDMS^E^).^13^, ^14^. HDMS^E^ allows the fragmentation of mobility‐separated precursor ions. KRAS standard peptides were analysed under identical conditions to those for the BSA standards and the drift times (bins) determined for each peptide. Only [M + 2H]^2+^ ions were selected for both calibrants and analyte ions. MS analyses were performed using HDMS^E^ for LC/MS and IMS/MS with continuous infusion. The parameters for the MS analysis were as described above, with TWIMS wave height of 40 V and IMS wave velocity of 1330 m/s.

### 2.5 Data analysis

TWIMS data were analysed using DriftScope software (Waters Corporation, Wilmslow, UK). LC/SRM data were analysed using Skyline. The unlabelled peptide retention times were confirmed by comparison with the heavy‐labelled standards which contained ^13^C_6_^15^N_2_‐lysine or ^13^C_6_^15^N_4_‐arginine at the C‐terminal, leading to a molecular mass increase of 8 or 10 Da, respectively. The unlabelled peptide peak areas were normalized to the heavy standard. The travelling wave CCS (^TW^CCS_N2_) was then calculated using a method described by Michaelevski et al.^15^

The drift times of each of the peptides were obtained from the raw data following ProteinLynx Global Server (PLGS; Waters Corporation, Wilsmlow, UK) processing of BSA peptides and KRAS mutant standards under the same MS conditions. The drift times of calibration ions and KRAS peptide ions were corrected to accommodate time spent outside the mobility region of the instrument using Equation (1):

(1) $$t\prime_{D}=t_{D}-\frac{\sqrt[{c}]{\frac{m}{z}}}{1000}$$

where *t*_D_ is the measured drift time of the precursor ion, *t*′_D_ is the corrected drift time of the precursor ion, *m*/*z* is the mass‐to‐charge ratio of the precursor ion and *c* is the enhanced duty cycle delay coefficient. A corrected DT was determined for BSA peptides and these times were plotted against known Ω′_N2_ values to establish the calibration line from the Bush Laboratory.^16^ Values of Ω′_N2_ for KRAS mutant peptide standards were then derived using the coefficients from the calibration line. The corresponding Ω_N2_ values (absolute ^TW^CCS_N2_) were obtained by factoring in the calculated reduced mass. IMS analysis for BSA and KRAS standards was ultimately performed using an IMS wave velocity of 1330 m/s.

## 3 RESULTS AND DISCUSSION

**Figure 1:**
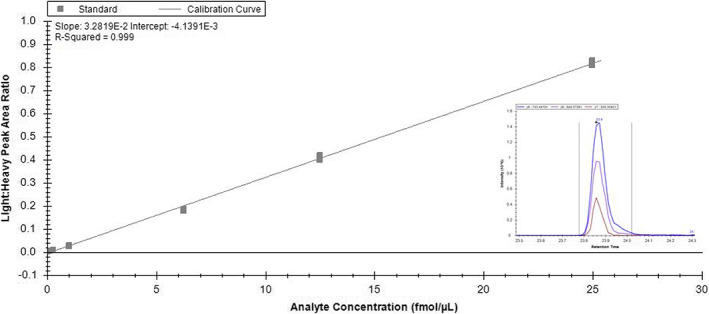
Representative calibration line from wild‐type 6–16 unlabelled peptide injected in triplicate and normalized to the heavy (^13^C_6_, ^15^N_2_) internal standard, and a representative Skyline chromatogram from 25 fmol/μL wild‐type unlabelled peptide standard

**Figure 2:**
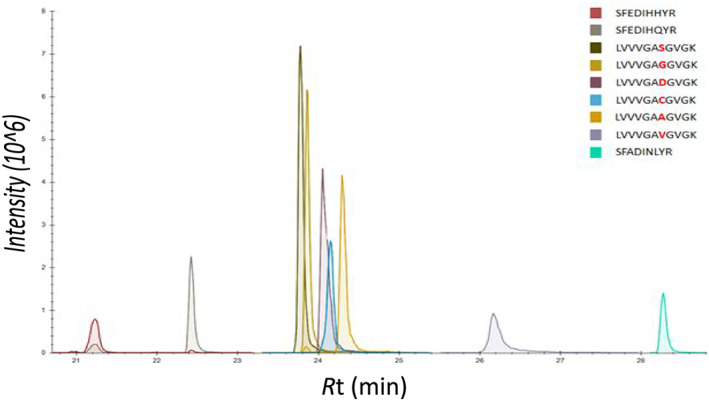
Chromatograms showing the separation of all nine analytes spiked in digested human plasma (WT, G12S, G12D, G12C, G12A and G12V 6–16 peptides and KRAS‐, NRAS‐ and HRAS‐specific peptides) with a 60 min reversed‐phase LC gradient using a Waters nanoACQUITY UHPLC system coupled to a Waters Xevo TQ mass spectrometer using LC/SRM

A method was required to identify the various KRAS mutations which are commonly found in different tumours including pancreatic, colorectal and lung cancers. Using LC/SRM, the wild‐type and five most common mutant peptides were separated using a 60 min LC gradient and accurately quantified using stable‐isotope‐labelled standards; the RAS isoforms were also detected. A representative chromatogram and a calibration line constructed in digested human plasma are shown in Figure 1, demonstrating the linearity of quantification of the wild‐type peptide. The separation of the RAS peptides using LC/SRM is shown in Figure 2. Four discrete retention time windows were used to enable the detection of 3–4 transitions for each peptide without compromising sensitivity. For each peptide the linear range was 0.125 to 25 fmol/μL, ensuring a sensitive assay for KRAS detection. Table 2 presents the lower limits of detection and quantitation for each of the peptides. A similar method has been developed by Wang et al^14^; however, this only detected the RAS‐specific peptides and RAS wild‐type, G12D and G12V peptides which are commonly found in pancreatic cancer. This LC/SRM method allows the potential for multiplex analysis of the whole RAS biology within biological samples.

**Table 2:** Calibration data from peptide calibration lines established in human plasma. Lower limit of detection (LLOD) and lower limit of quantitation (LLOQ) values for each peptide when measured using LC/SRM^a^

Peptide sequence	Retention time (min)	LLOD (fmol/μL)	LLOQ (fmol/μL)	*R* ^2^
SFEDIHQYR	22.50	0.31	1.0	0.998
SFEDIHHYR	21.30	0.50	1.0	0.996
LVVVGAGGVGK	23.95	0.21	1.0	0.999
LVVVGAAGVGK	24.40	0.52	12.5	0.998
LVVVGASGVGK	23.87	0.24	1.0	0.998
LVVVGAVGVGK	26.23	2.75	6.25	0.998
LVVVGADGVGK	24.16	0.27	1.0	0.999
LVVVGACGVGK	24.23	0.31	1.0	0.998
SFADINLYR	28.37	0.24	6.25	0.998

LLOD and LLOQ are derived from Skyline using linear regression with ratio of heavy to light normalisation and for LLOD blank plus 2 × SD. Replicate coefficients of variation all below 5% for all concentrations except 0.125 fmol/μL which did not exceed 15% for any of the peptides.

**Figure 3:**
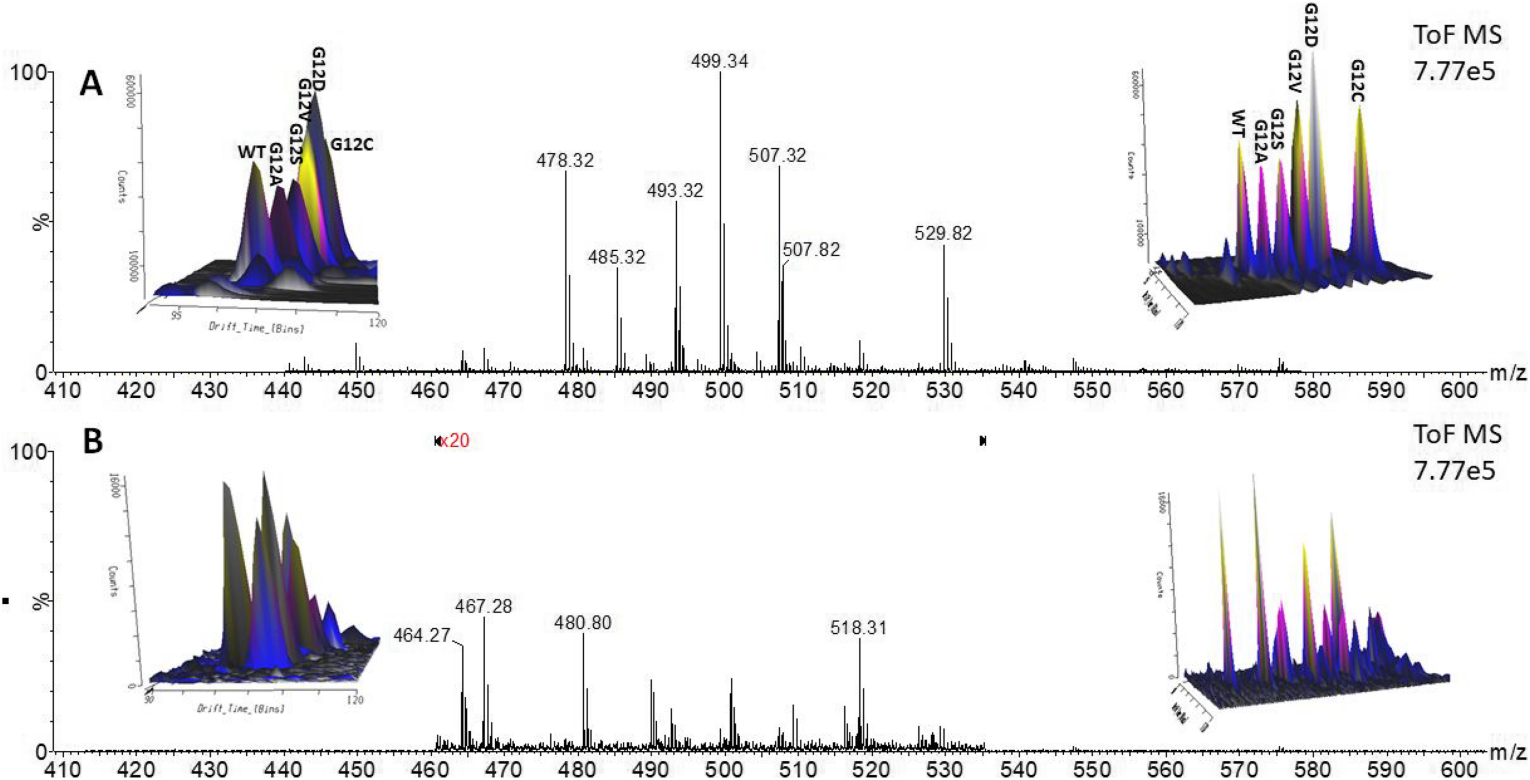
KRAS 6–16 peptide mix (wild‐type and the five most common mutations) spiked (500 fmol/μL) into tryptically digested human plasma peptides analysed using IMS/MS via continuous infusion (1 μL/min). (A) MS spectrum obtained by extracting spectra from relevant region of IMS acquired data. Inset (left) shows mobility separated peptide ions whilst inset (right) is the same mobilogram but orientated approximately 90°. (B) Identical analysis of tryptically digested plasma in the absence of the KRAS 6–16 peptide mix. The extracted MS spectra show clear absence of interfering *m*/*z* peaks. Note: relevant region has been amplified 20‐fold

In certain settings it may be appropriate to provide qualitative data which simply inform the clinician as to which mutation(s) are present. Therefore, analysis with IMS/MS using continuous infusion provides a potentially rapid capability of phenotyping patient samples. TWIMS allows highly specific identification of mutations in KRAS because the various mutant peptides can be separated by mobility in addition to the *m*/*z* value. Figure 3A shows the separation of the peptides spiked into digested human plasma in a three‐dimensional ion mobility spectrum. Due to the infusion aspect of the IMS experiment, we can rapidly detect the different mutations of KRAS. Figure 3B shows the same analysis of digested human plasma without the peptides and it should be noted that the scale is 37.5 times greater in the KRAS peptide mobilogram. The MS spectra and mobilograms thus demonstrate that the ion mobility provides sufficient specificity that is distinct from background signals. In addition to the *m*/*z* values, we can obtain ^TW^CCS_N2_ values that are specific to the mutated peptides. Table 3 presents the ^TW^CCS_N2_ values ultimately derived from the drift time measurement of the individual peptides. The ^TW^CCS_N2_ values obtained for each peptide reflected *m*/*z* except for G12V which had a larger ^TW^CCS_N2_ than G12D despite having a smaller *m*/*z value.* This is presumably because of the aliphatic nature of valine which might increase the ^TW^CCS_N2_ of the peptide. The measurement of ^TW^CCS_N2_ could be used to accurately determine the mutation status in a clinical or biological sample.

**Table 3:** Detection of KRAS 6–16 peptide mix. Detection of individual KRAS peptides and the resultant ^TW^CCS_N2_ estimations using a digested BSA peptide calibration line^a^

KRAS variant	Sequence	MI mass	Measured *m*/*z*	Average DT bins	DT	DT′	Red. mass	Ω′ (Å^2^)	Calculated ^TW^CCS_N2_ (Å^2^)
WT	LVVVGA**G**GVGK	954.5862	478.2959	100.39	6.927	6.894	5.216	856.91	328.5
G12A	LVVVGA**A**GVGK	968.6019	485.3037	103.86	7.166	7.133	5.218	871.43	334.0
G12S	LVVVGA**S**GVGK	984.5968	493.3012	105.68	7.292	7.259	5.219	879.03	336.9
G12V	LVVVGA**V**GVGK	996.6332	499.3194	107.69	7.430	7.397	5.220	887.42	340.0
G12D	LVVVGA**D**GVGK	1012.592	507.2986	107.23	7.399	7.365	5.221	885.49	339.2
G12C	LVVVGA**C**GVGK	1057.595	529.8005	109.22	7.536	7.501	5.224	893.77	342.2

MI mass, monoisotopic mass; measured *m*/*z*, acquired *m*/*z* of the peptide; average drift time bins (*n* = 3); DT, drift time; DT′, corrected drift time; red. mass, reduced mass; Ω′, normalized CCS value; ^TW^CCS_N2_, CCS value derived in travelling wave ion mobility in nitrogen gas.

The ^TW^CCS value is a constant and can be easily measured using TWIMS (the drift times were obtained as an average of three determinations with a coefficient of variation across the measurements of 0.14%). LC/MS is potentially susceptible to shift in the chromatographic retention times which, when looking for a single mutation, could potentially affect selectivity. These retention time shifts can of course be ameliorated by the use of standards. However, where there is chromatographic overlap in transition windows, even small retention time shifts may hinder the reliable measurement of peptides and thus the translation of the method into clinical laboratories. TWIMS offers considerably reduced analysis time. Instead of using the long chromatographic gradients required for sufficient liquid‐phase separation, rapid profiling of samples could be achieved in several minutes using TWIMS, enabling a selective and specific method for the higher throughput analysis of samples. Sensitivity may be a disadvantage for TWIMS but the continual improvement in capture technologies and strategies will greatly enable clinical implementation of TWIMS and other IMS/MS approaches.

## 4 CONCLUSIONS

The ability of TWIMS to differentiate the different KRAS mutant peptides demonstrates the distinct opportunity for this technology to provide a highly selective and specific method that can precisely discriminate KRAS mutants and thus allow the phenotyping of biological samples. It is demonstrated that, using previously generated ^TW^CCS_N2_ calibration values, we could accurately determine the ^TW^CCS_N2_ values of each of the KRAS mutant peptides for the first time. In addition, we have described a LC/SRM method that can allow quantitation of these clinically important KRAS mutations.
